# Fabrication and examination of polyorganophosphazene/polycaprolactone-based scaffold with degradation, in vitro and in vivo behaviors suitable for tissue engineering applications

**DOI:** 10.1038/s41598-022-18632-8

**Published:** 2022-11-01

**Authors:** Khodayar Gholivand, Mahnaz Mohammadpour, Seyed Alireza Alavinasab Ardebili, Rahime Eshaghi Malekshah, Hadi Samadian

**Affiliations:** 1 Department of Chemistry, Faculty of Sciences, Tarbiat Modares University, Tehran, Iran; 2 Medical Biomaterial Research Centre (MBRC), Tehran University of Medical Sciences, Tehran, Iran; 3 Research Center for Molecular Medicine, Hamadan University of Medical Sciences, Hamadan, Iran

**Keywords:** Biochemistry, Biotechnology, Computational biology and bioinformatics, Chemistry

## Abstract

The present study aimed to synthesis a proper scaffold consisting of hydroxylated polyphosphazene and polycaprolactone (PCL), focusing on its potential use in tissue engineering applications. The first grafting of PCL to poly(propylene glycol)phosphazene (PPGP) was performed via ROP of ε-caprolactone, whereas PPGP act as a multisite macroinitiator. The prepared poly(propylene glycol phosphazene)-graft-polycaprolactone (PPGP-*g*-PCL) were evaluated by essential tests, including NMR, FTIR, FESEM-EDS, TGA, DSC and contact angle measurement. The quantum calculations were performed to investigate molecular geometry and its energy, and HOMO and LUMO of PPGP-*g*-PCL in Materials Studio2017. MD simulations were applied to describe the interaction of the polymer on phospholipid membrane (POPC128b) in Material Studio2017. The C2C12 and L929 cells were used to probe the cell–surface interactions on synthetic polymers surfaces. Cells adhesion and proliferation onto scaffolds were evaluated using FESEM and MTT assay. In vitro analysis indicated enhanced cell adhesion, high proliferation rate, and excellent viability on scaffolds for both cell types. The polymer was further tested via intraperitoneal implantation in mice that showed no evidence of adverse inflammation and necrosis at the site of the scaffold implantation; in return, osteogenesis, new-formed bone and in vivo degradation of the scaffold were observed. Herein, in vitro and in vivo assessments confirm PPGP-*g*-PCL, as an appropriate scaffold for tissue engineering applications.

## Introduction

Tissue engineering is based on the triad combination of living cells, extracellular matrix (ECM)-mimicking scaffold and factors growth employed to regenerate and repair defective tissue^1–3^. A functional scaffold should be implantable into the tissue defect site, biodegradable, mechanically robust, compatible with the surrounding cells and tissues, and should have non-toxic degradation products^1–4^. A proper scaffold improves the proliferation and differentiation of the cells, thus facilitating the regeneration and repair of damaged tissue. For this purpose, the use of biocompatible polymeric materials as a scaffold extensively increased^2,4^.

Polymeric scaffolds in two types of natural and synthetic are made and examined. Natural polymers (such as hyaluronic acid, chitosan, collagen, and alginate) have great potential for being used in biological applications. These polymers exhibit excellent biocompatibility, unique biodegradable, high water absorption capacity, low-toxic degradation products, and great similarity to the extracellular matrix (ECM)^1,5–7^, but their use is limited due to high cost, structural complexity, complicated extraction, and sensitivity to contamination and harsh chemical reactions^7,8^. The synthetic polymeric scaffolds were designed and synthesized with variant functional groups, and these polymers possess various morphologic characters, controllable degradability, and better mechanical strength were fabricated and rapidly expanded. The synthetic polymers like poly(lactic acid), poly(glycolic acid), poly(caprolactone), poly(propylene fumarate), and their copolymers are commonly used for tissue repair and regeneration^5,6,8,9^. However, they are associated with drawbacks like an inflammatory biological response, poor cellular attachment, poor bioactivity, and toxicity^6,8^.

Cell behavior (adhesion, proliferation and differentiation) on the scaffold strongly depends on scaffold features such as porosity, wettability, surface charge, electroactivity, softness, stiffness, etc^10–12^.

Polycaprolactone (PCL) for various reasons—for instance, its physical and biological properties, tissue compatibility, degradation into non-toxic products, and resorbability of by-product—is one of the most commonly used synthetic polymers in tissue engineering^4,13,14^. The PCL is a rubbery semicrystalline polyester (T_g_ ~  − 60 °C and T_m_ ~ 60 °C) and sensitive to hydrolysis (due to the presence of aliphatic ester bonds) under physiological conditions. Also, it is soluble in some organic solvents like dichloromethane, chloroform, toluene, cyclohexanone and has been approved by the FDA^4,13–15^. In polyester compounds, the aliphatic chain length affects degradation rate, wettability, hydrophilicity, and so forth^16–18^. The PCL with five methine groups is a hydrophobic polyester with undesired wettability properties, poor cell adhesion and prolonged degradation rate, so not very suitable for tissue engineering applications^4,14,16,19^. In order to remove limits of PCL scaffolds, various strategies have been employed, including synthesis of polymer blends, copolymers and hybrid polymers, surface physical/chemical modifications, using different polymerization techniques^4,13,14^.

Currently, via the substitution reactions of chlorine atoms along inorganic poly(dichlorophosphazene) P‒N backbone with diverse organic amines or alkoxides were achieved a wide array of poly(organophosphazenes), that are hybrid organic–inorganic macromolecules, and organic side groups determine the physical–chemical properties, structural characteristics and performance fields of synthesized poly(organophosphazenes). If polyphosphazenes P‒N backbone is substituted with proper sides groups such as amino acids and their derivatives, 4-hydroxybenzoate, etc., causing the polymer to become unstable and degradable at hydrolytic conditions. The degradation rate under these conditions is much-influenced type and ratio of the chain pendant groups. It is important to note that polymer chain decomposition due to hydrolysis reaction generates non-toxic (or low-toxic) degradation byproducts, namely phosphate, ammonia and corresponding side groups^20–23^. In recent years, biodegradable polyphosphazenes containing biocompatible side groups—which have suitable adaptability with cells and tissues—were considered for tissue engineering applications and obtained promising results^21–23^. Aiming to obtain special and functional properties, a number of chain polyphosphazenes have been grafted with other linear polymers, such as oligopeptides, polyoxazolines, polyphenylene vinylene, polystyrene, polycaprolactam, and etc. As mentioned, the pure PCL cannot meet demands of some of the bio-medical applications E.g. bone tissue engineering, for its weakness in degradability, mechanical properties and hydrophobicity, so PCL alone may not be a satisfactory substance for the development of bone substitutes^21,22^.

Over the past several years, hydroxylated polyolefins (polypropylene or polyethylene) have been used as macro initiators in the ROP of lactones to the synthesis of polyesters-based block and graft polymers with unique biological and mechanical properties^24,25^. In this study, In order to build a biocompatible scaffold with desirable properties of PCL, without its common limitations, we synthesized a new graft polymer, poly(propylene glycol phosphazene)-*graft*-polycaprolactone (PPGP-*g*-PCL), from a water-soluble hydroxylated polyphosphazene, named poly(propyleneglycol phosphazene) (PPGP), and caprolactone monomers under the conditions required for ring-opening polymerization of lactones and formation of PCL, in which PPGP plays the role of multisite macroinitiator.

The polymers were identified by ^1^H, ^13^C and ^31^P NMR, FT-IR spectroscopies, and analyses of FESEM-EDS and thermal. Static water contact angle of novel PPGP-*g*-PCL was compared with pure PCL to monitor the change in fabricated polymer hydrophobicity/hydrophilicity. Aiming to predict the polymer’s molecular structure and energy and estimate HOMO and LUMO orbital energies, a quantum computation was performed by the DMol^3^ module based on DFT-D correction. Then, the interactions between the polymer and phospholipid membrane (POPC128b) were monitored using MD simulations.

Afterward, to investigate the potential use of the polymer for defective organs regeneration and tissue engineering, we evaluated cell adhesion, proliferation, differentiation and cytotoxicity for cultured cells on the polymeric scaffolds made from PPGP-*g*-PCL and its blend with pure PCL.

In the end, to further evaluate the biosafety of our graft polymer and the blend polymers, in vivo evaluation was performed by intraperitoneal implantation of polymeric scaffolds in BALB/c mice. Eventually, the tissue reactions were analyzed by Hematoxylin and eosin (H-E), Masson's trichrome (TRI), alizarin red and methylene blue stains and then microscopic observations, and computer software.

## Results and discussion

### Synthesis and characterization of polymers

**Figure 1:**
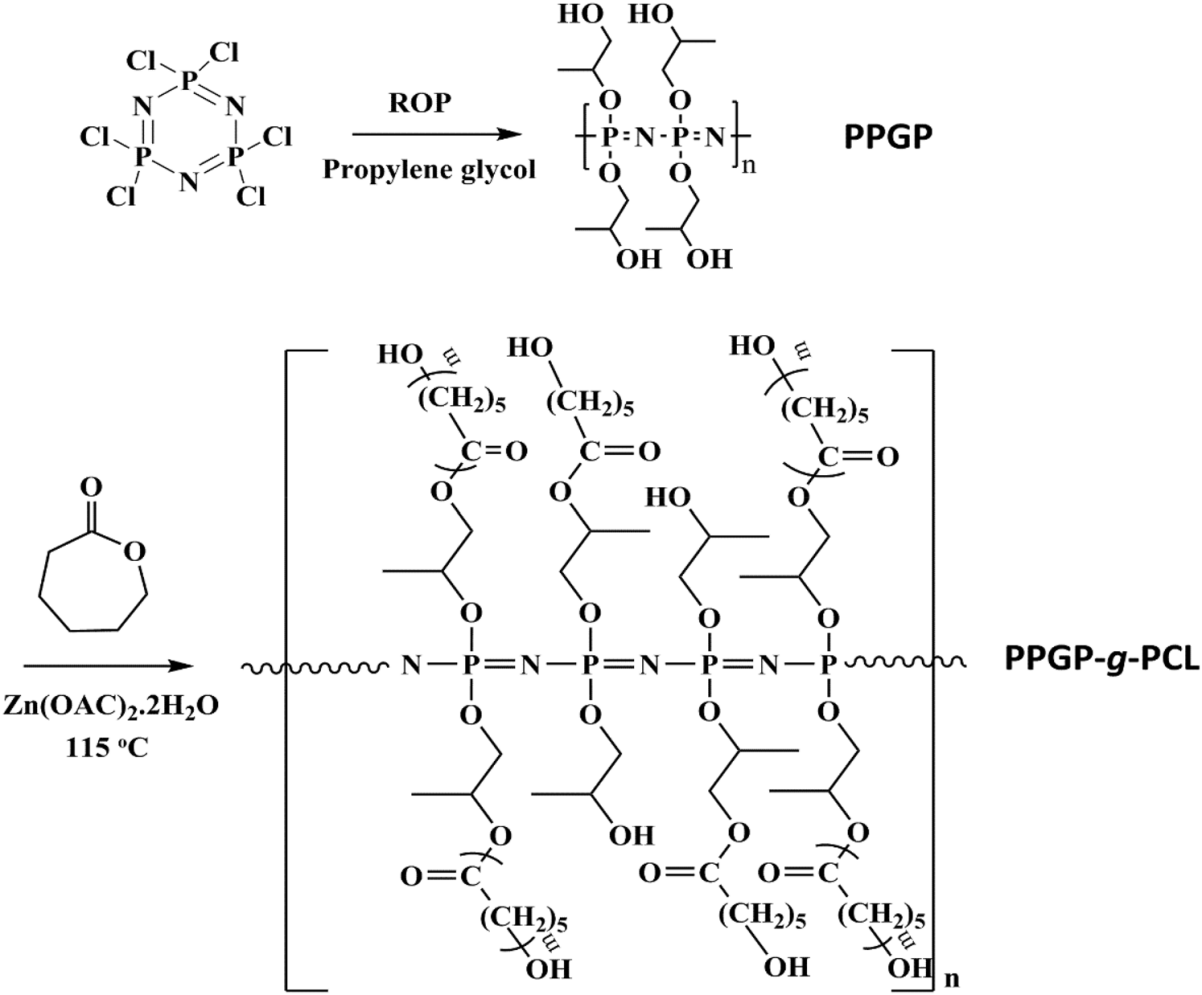
Synthesis and structures of PCL-grafted poly(organophosphazene) through ROP of caprolactone using PPGP multisite initiator.

For synthesize of poly(propyleneglycol)phosphazene-*graft*-polycaprolactone (PPGP-*g*-PCL), we produced the phosphazene random polymer carrying terminal alcohol functions, labeled with PPGP, as the water-soluble precursor^26^. Grafting of PCL was conducted via ROP of *ε*-CL in present Zn(AC)_2_.2H_2_O catalyzer by pendant hydroxyl groups of the PPGP precursor as multisite macroinitiator^27^. This concept is illustrated in Fig. 1. Obtained graft polymer is soluble in ethanol and insoluble in water. The purity and composition of the obtained PPGP-*g*-PCL graft polymer were assessed by analyses of NMR, FTIR, FESEM-EDS, TGA, DSC and contact angle measurement. Meanwhile, the molecular geometry of PPGP-*g*-PCL and its energy, as well as HOMO and LUMO, was calculated using by DMol^3^ module based on DFT-D correction.

### NMR spectroscopy

**Figure 2:**
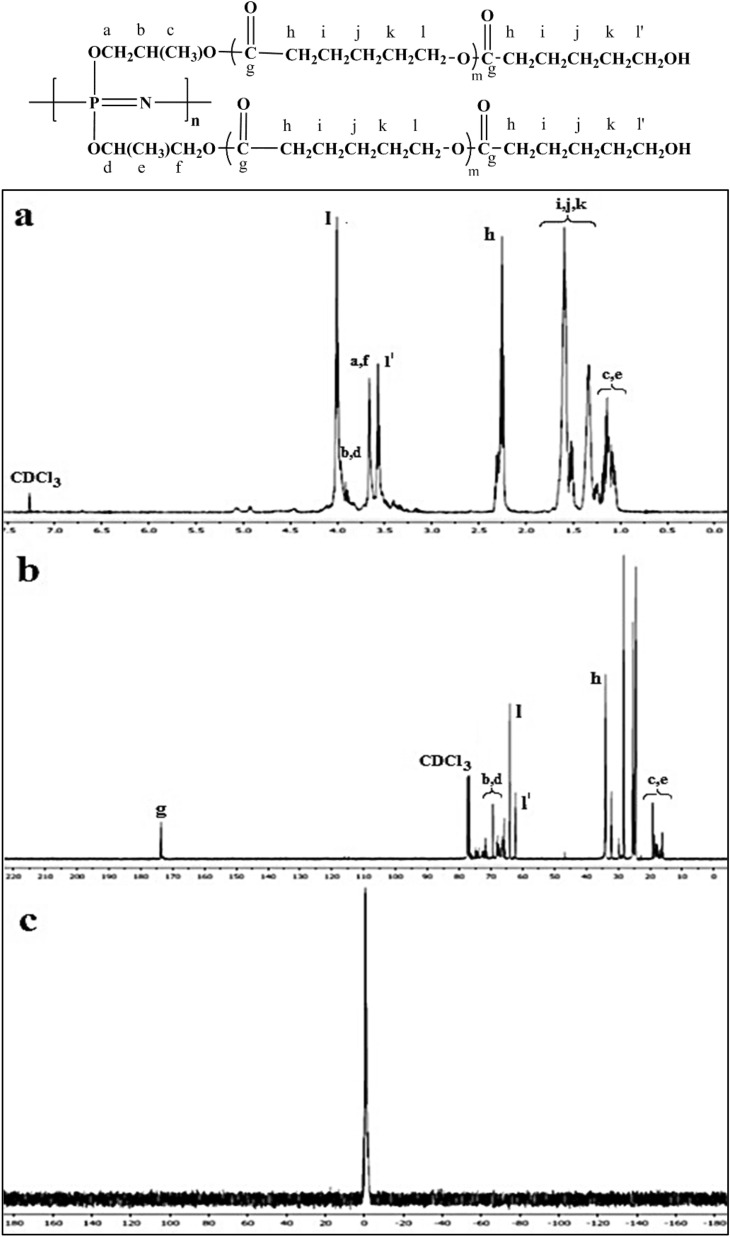
(**a**) ^1^H NMR, (**b**) ^13^C NMR and (**c**) ^31^P NMR of PPGP-*g*-PCL graft polymer in CDCl_3_-d^1^ solvent.

^31^P NMR analysis of the hydroxyl-containing PPGP showed a broad signal at around − 1 ppm, while there were no P–Cl peaks related to cyclophosphazene and or polydichlorophosphazene^28^. The nature of the primary and the secondary hydroxyl groups on propylene glycol and comparison of ^1^H NMR spectra of propylene glycol and PPGP indicated that propylene glycols were randomly bonded to the polymer backbone. ^1^H NMR spectrum of the PPGP showed a collection of peaks between 3.24 and 3.77 ppm attributable to the methylene and methine protons, while a collection of chemical shifts between 0.98 and 1.18 was related to methyl protons of propylene glycols attached to the polymer chains. Figure 2c demonstrates the ^31^P NMR spectrum of PPGP-*g*-PCL, which can be observed only the one broad signal at − 1 ppm related to P atoms along the polymer. ^1^H NMR and ^13^C NMR spectrums of PPGP-*g*-PCL confirm ring-opening polymerization of *ε*‐caprolactone by the terminal OH groups of PPGP (Fig. 2a,b). Regarding propyleneglycol randomly via 1̊ or 2̊ OH groups bonded to P atoms, in the ^1^H NMR spectrum of PPGP-*g*-PCL, resonances at 3.88–3.93 ppm, 3.66 ppm, and 1.04–1.19 ppm are corresponding to CH, CH_2_, and CH_3_ groups of propyleneglycol, respectively. In addition, an intensive resonance signal at 4.00 ppm is the assignment for protons of CH_2_–O–CO and an intensive signal at 3.58 ppm is related to methylene protons in CH_2_–OH end groups of grafted polycaprolactone. Also, the peak assigned to the protons of O–CO–CH_2_ groups appeared as a triple at 2.25 ppm. Two sets of multiplet resonance signals at around 1.51–1.67 ppm and 1.29–1.40 ppm are attributed to the other methylene groups on the polycaprolactone chain^27,29^.

In the ^13^C NMR spectrum in Fig. 2b no peaks appeared at 175 ppm assigned to the carbonyl carbon of *ɛ*-caprolactone while the resonance signal at 173.52 ppm is assigned to the carbonyl of polycaprolactone. The resonance signals related to CH_2_–O–CO are located at around 64.08 ppm, CH_2_–OH at 62.38 ppm, and O–CO–CH_2_ at 34.07 ppm. The peaks set at 69.41 and 68.22 ppm are related to CH groups (O–CH (CH_3_)–CH_2_‒O–CO and CH_2_–CH (CH_3_)–O‒CO), and signals at 19.24 and 16.70 ppm are related to CH_3_ groups. Other peaks set are related to CH_2_ groups of propyleneglycol and polycaprolactone^27,30^.

### FTIR spectroscopy

**Figure 3:**
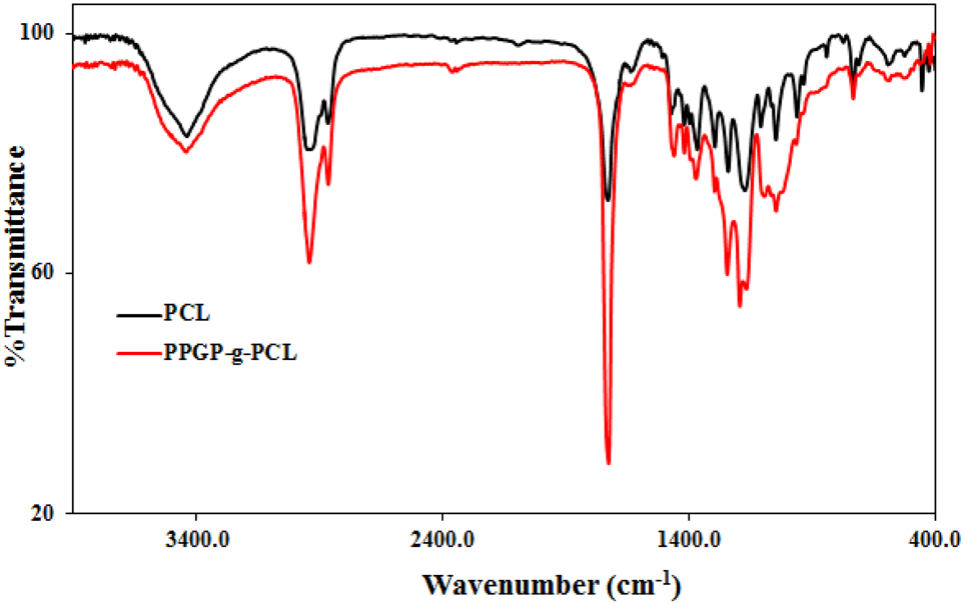
FT-IR patterns of PPG-*g*-PCL compared with PCL.

The results obtained by FTIR analysis are in agreement with those obtained by the ^1^P, ^1^H and ^13^C NMR spectrum. In Fig. 3, FTIR spectroscopy PPGP-*g*-PCL was compared with PCL for further studies on the chemical structure of the new synthetic polymer. For PCL, peaks at 3439.82 cm^−1^ are due to vibrations of hydroxyl groups and absorbed water. Characteristic absorbed bands at 2950.45 cm^−1^ and 2866.83 cm^−1^ correspond to symmetric and asymmetric vibrations of CH_2_ groups. The stretching vibration of ester carbonyl groups (–C=O) appeared at 1728.47 cm^−1^ and adsorption bands at 1471 cm^−1^, 1419 cm^−1^ and 1366 cm^−1^ are assigned to CH_2_ bending vibrations. The spectra also contain bands at 1294 cm^−1^ related to C‒O and C‒C stretching, 1240.44 cm^−1^ due to C‒O‒C stretch asymmetric, and 1172.86 cm^−1^ attributed to C‒O‒C bond^29,31–33^. In the FTIR spectrum of PPGP, stretching vibration of P‒N bond and P=N double bond are located at 869 cm^−1^ and 1209 cm^−1^, respectively. The peak at 1056 cm^−1^ is attributed to P‒O‒C bonds of PPGP. FTIR spectrum of PPGP-*g*-PCL contains all absorbance peaks of PCL and PPGP, but due to overlap of its absorbance peaks with PCL and PPGP, comparison of spectrums cannot easily be done. However, vibration bands at 1193.25 cm^−1^ derived from the P=N band and shift in observed wavelengths are the difference between PPGP-*g*-PCL with PCL and PPGP^20,21,27^. ^1^H-NMR and FTIR results clearly indicate the presence of the pre-assumed structure.

### TGA analysis

**Figure 4:**
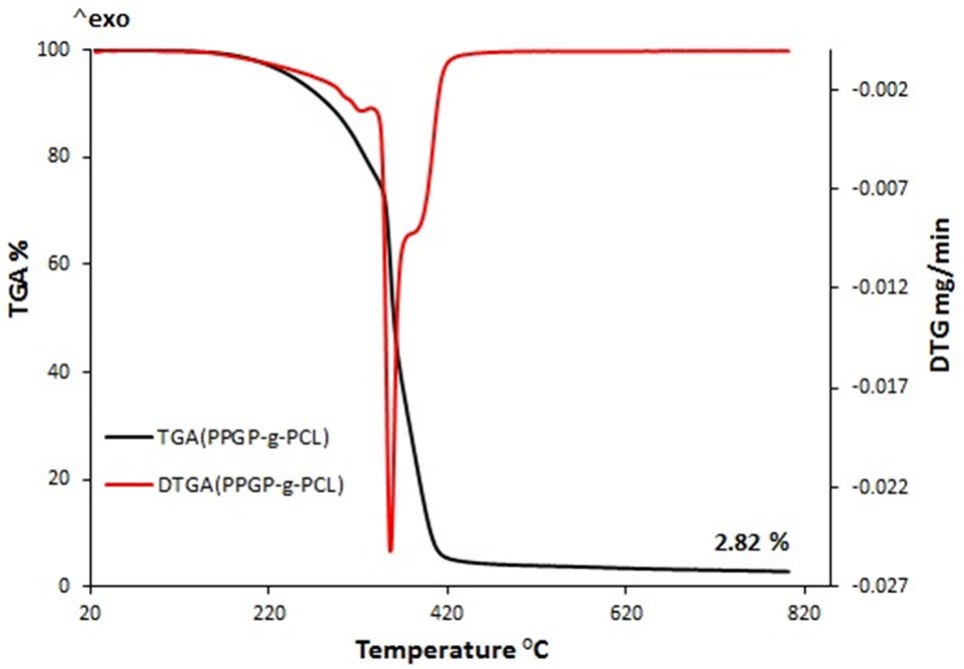
Thermogravimetric analysis (TGA) of the synthesized polymer. The red line shows the first derivative of the TGA curve.

Thermogravimetric analysis (TGA) and differential scanning calorimetry (DSC) measurements were employed for the investigation of thermal properties of PPGP-*g*-PCL graft polymer. According to Fig. 4, it was estimated that the initial slight drop below 150 °C was related to the evaporation of the remaining solvent and the adsorbed moisture. The weight loss observed between 150 °C to about 300 °C is due to the rupture of the polymeric chains and, following that, thermal decomposition^34,35^. Continuous pyrolysis with a faster degradation rate centered at 350 °C is attributed to the dissociation of organic units and their evaporation and generation of cyclophosphazene oligomers arising from backbone depolymerization^27,34–36^. The gradual drop at near 400 °C is associated with the release of the formed cyclophosphazenes in the previously step.

### DSC analysis

**Figure 5:**
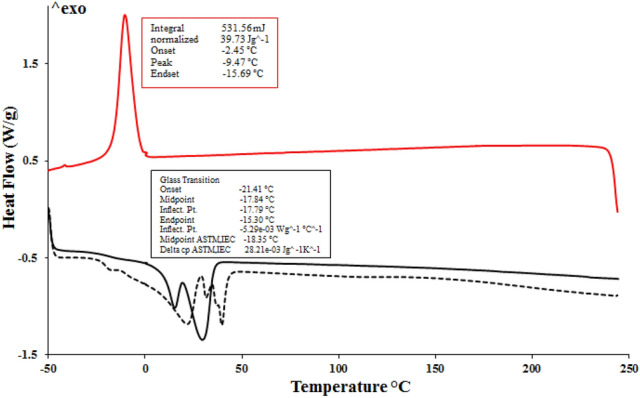
The differential scanning calorimetry (DSC) curves recorded during the PPGP-*g*-PCL curing; all runs obtained at a 10 °C min^−1^ heating rate.

To observe the melting and crystallization behavior of synthetic PPGP-*g*-PCL polymer, DSC studies were conducted from − 70 to 300 °C under a nitrogen atmosphere. The thermal profile of PPGP-*g*-PCL in Fig. 5, revealed a glass transition temperature midpoints (T_g_) of − 17.84 °C and two endothermic peaks located at 15.45 and 29.54 °C, which corresponded to the melting temperatures (T_m_). The appearance of two T_m_ peaks in the heating curve represents the presence of variable-length arms in the polymer^35,37^. The cooling curve of the DSC thermogram exhibits an exothermic peak at − 9.47 °C ascribed to the crystallization temperature. The data show that the PPGP-*g*-PCL, like PCL, is a semicrystalline polymer, whereas it was observed that PPGP (due to the lack of T_c_) was amorphous (Fig. S1).

### FESEM-EDS

**Figure 6:**
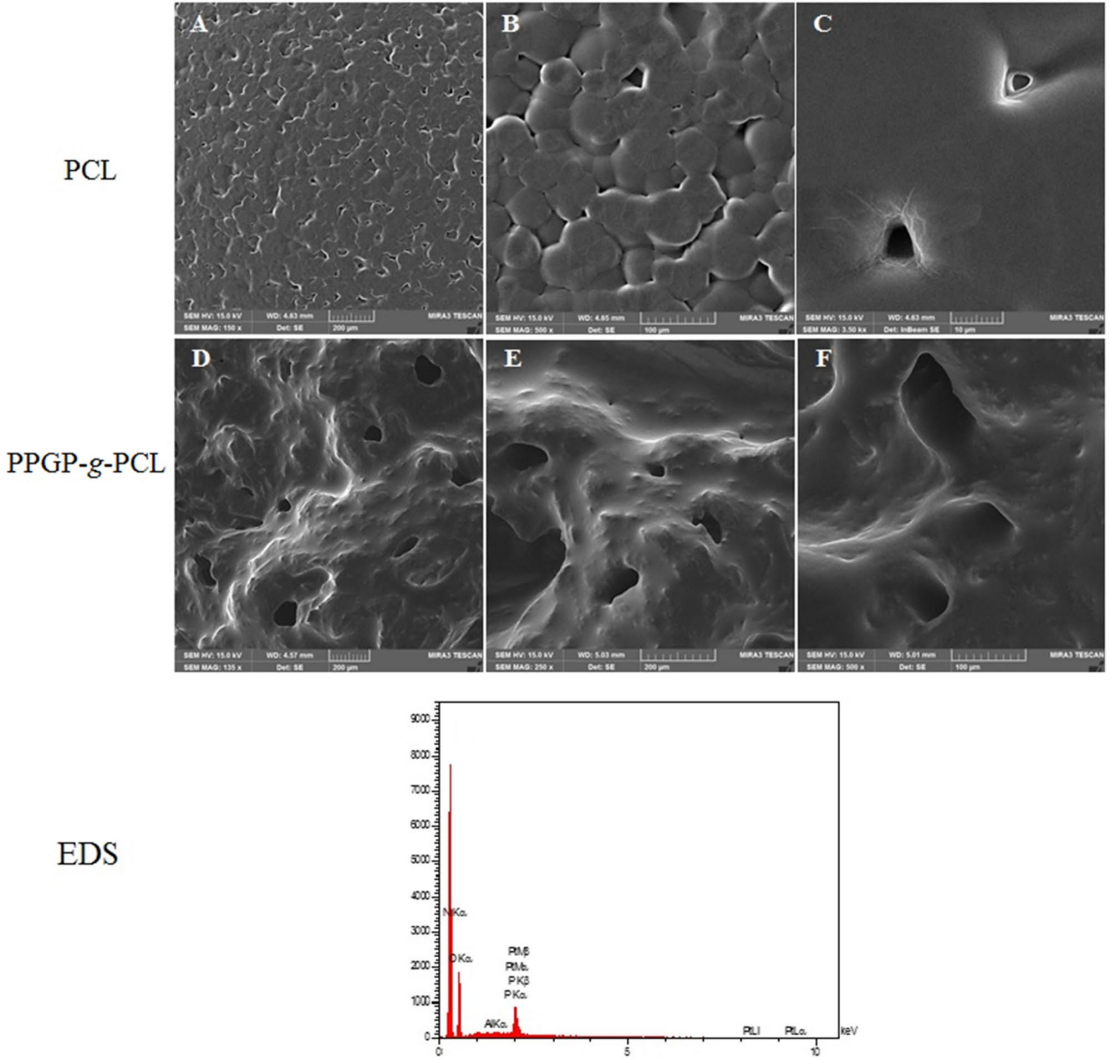
FESEM images of the porous surfaces of pure PCL and synthesized PPGP-*g*-PCL and the EDS results for the PPGP-*g*-PCL. PPGP-*g*-PCL samples exhibited a greater number of microporous structures than PCL surfaces.

We assessed the morphology and composition of prepared PPGP-*g*-PCL surface by field-emission scanning electron microscopy (FESEM) and energy dispersive spectroscopy (EDS) analysis for their microstructure and elemental analysis in Fig. 6. The PPGP-*g*-PCL exhibited a homogeneous and uniform surface with proper porosity that is beneficial in order to promote cell nutrition, proliferation, migration and differentiation^3,6,38^. EDS mapping demonstrated the element's content in the surface of the sample. Sharp peaks for C and O, which are the main elements of linked propylene glycol and PCL, can be seen in the figure. The peaks of P and N with less intense EDS spectra agree with the PPGP-*g*-PCL predicted structure. No extra peaks are seen on the spectra.

### Contact angle measurement

**Figure 7:**
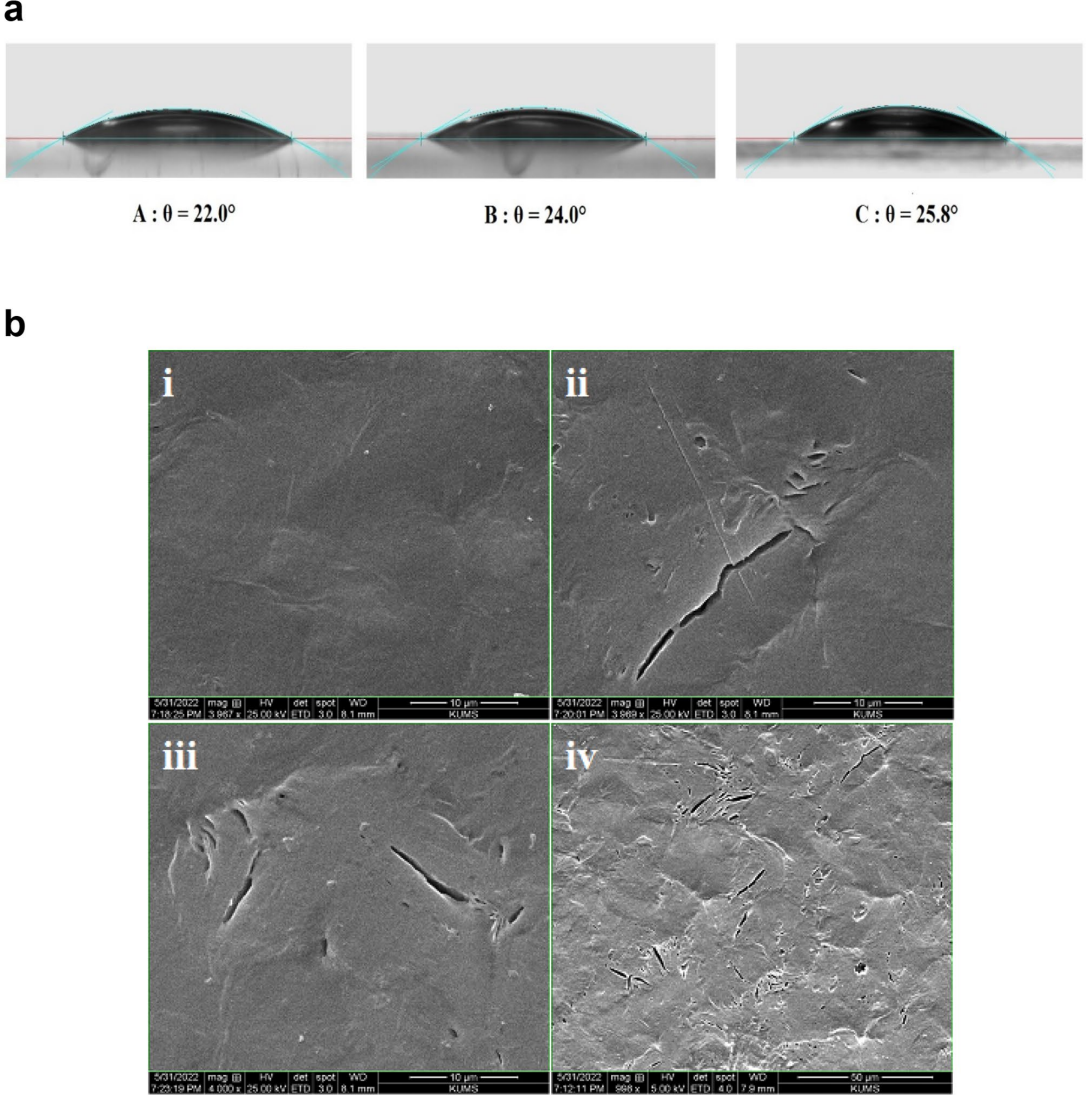
(**a**) Representative photographs of contact angle of a water droplet on the graft polymers: (A) PPGP-*g*-PCL, (B) PCL/PPGP-*g*-PCL (30/70 wt%) and PCL/PPGP-*g*-PCL (40/60 wt%). (**b**) SEM images of (i) PCL, (ii‒iv) PCL80%/PPGP-g-PCL20% blend polymer after a ten-day degradation analysis. ((ii), (iii) = magnifications of 10 μm and (iv) magnifications of 50 μm).

Scaffold surface wettability (hydrophobicity and hydrophilicity properties), through controlling the amounts and the types of proteins that are adsorbed on the surface, seriously influence the rate of cell proliferation, spreading, and differentiation^2,12,16,38^. PCL/PPGP-*g*-PCL (60/40 wt%) and PCL/PPGP-*g*-PCL (70/30 wt%) blends were prepared using physical blending. The surface wetting properties of PPGP-*g*-PCL, PCL/PPGP-*g*-PCL (40/60 wt%) blend, and PCL/PPGP-*g*-PCL (30/70 wt%) blend was evaluated via water contact angle (WCA) measurements by sessile drop technique at room temperature. Drops of ultra-pure water were deposited on each scaffold, and immediately the contact angle between water drop and solid substrate was determined. Images are displayed in Fig. 7a. The surface of the PPGP-*g*-PCL polymer with a mean contact angle of 22° showed high degrees of hydrophilicity. The PCL is hydrophobic in nature^2,4,39^ but after mixing with PPGP-*g*-PCL showed a dramatic change from a hydrophobic surface to a hydrophilic surface, with angles of 25.8° (40/60 wt% blend) and 24° (30/70 wt% blend). This could be due to the change in roughness, polarity, or the number of hydrogen bonds on the surface^39^. So, the observations of the hydrophilicity of the PCL can be significantly optimized by mixing with miscible PPGP-*g*-PCL hydrophilic polymer.

### Mechanical properties and degradability behavior of the fabricated scaffolds

A convenient scaffold for bone tissue engineering must have admissible mechanical properties to be able to withstand the stresses when it is evaluated in vitro or implanted in vivo. The mechanical strength of the produced constructs was assessed by a compression test (Table 1), and the obtained results show that the compressive property of PCL with a compressive strength of 1900 MPa is higher than that of a sample containing PPGP-g-PCL with a compressive strength of 1208.8 MPa. The revers correlation is seen between the existence of PPGP-g-PCL and the compressive strength, which can be related to the porosity created by PPGP-g-PCL. Similar results were also received by other researchers when PCL was blended with PLLA and or PLGA. Moreover, this was consistent with previously reports that PCL-based scaffolds show a revers relationship between the mechanical strength and the pore volume fraction^2,6^.

**Table 1:** Data of compressive mechanical test on PCL and PCL80%/PPGP-g-PCL blend polymers.

Sample	Compressive strength (MPa)	Young’s modulus (MPa)	Elongation at break
PCL	1900	8313.83	32.248
PCL/PPGP-g-PCL (80:20)	1208.8	6101.35	12.83

Biodegradation of a scaffold is a determining factor in its qualification to tissue engineering requests and have a pivotal role in bone tissue rebuilding. Degradation of scaffold provides suitable space for cell migration and proliferation, angiogenesis and new tissue formation. On the other, high degradation rate restricts cell adhesion and migration and causes tissue defects at the implant site. PCL, as a polyester with five CH_2_ groups, is a stable polymer and undesirable in terms of degradation rate when used alone as a scaffold^4,14,16,19^. We therefore evaluated the degradation of pure PCL and PPGP-g-PCL scaffolds and PCL/PPGP-g-PCL mix immersed in solutions of PBS and AcOH buffer. As shown in Fig. S3, PPGP-g-PCL offers the highest degradation grade in both media, that according to the hydrophilicity of this polymer, it can be due to its greater interaction with water molecules through ionic interactions and hydrogen bonding. PCL shows a very low degradation rate, but its degradation amount expands when mixed with PPGP-g-PCL. Considering that the degradation of these polymers is done by hydrolysis mechanism, the distribution of PPGP-g-PCL as a degradable medium at PCL leads to easier access of water molecules to the scaffold and increases its degradation degree. Moreover, SEM images after acid degradation show ablation arising from hydrolysis of scaffolds (Fig. 7b (i‒iv**)**.

### Computational method

**Figure 8:**
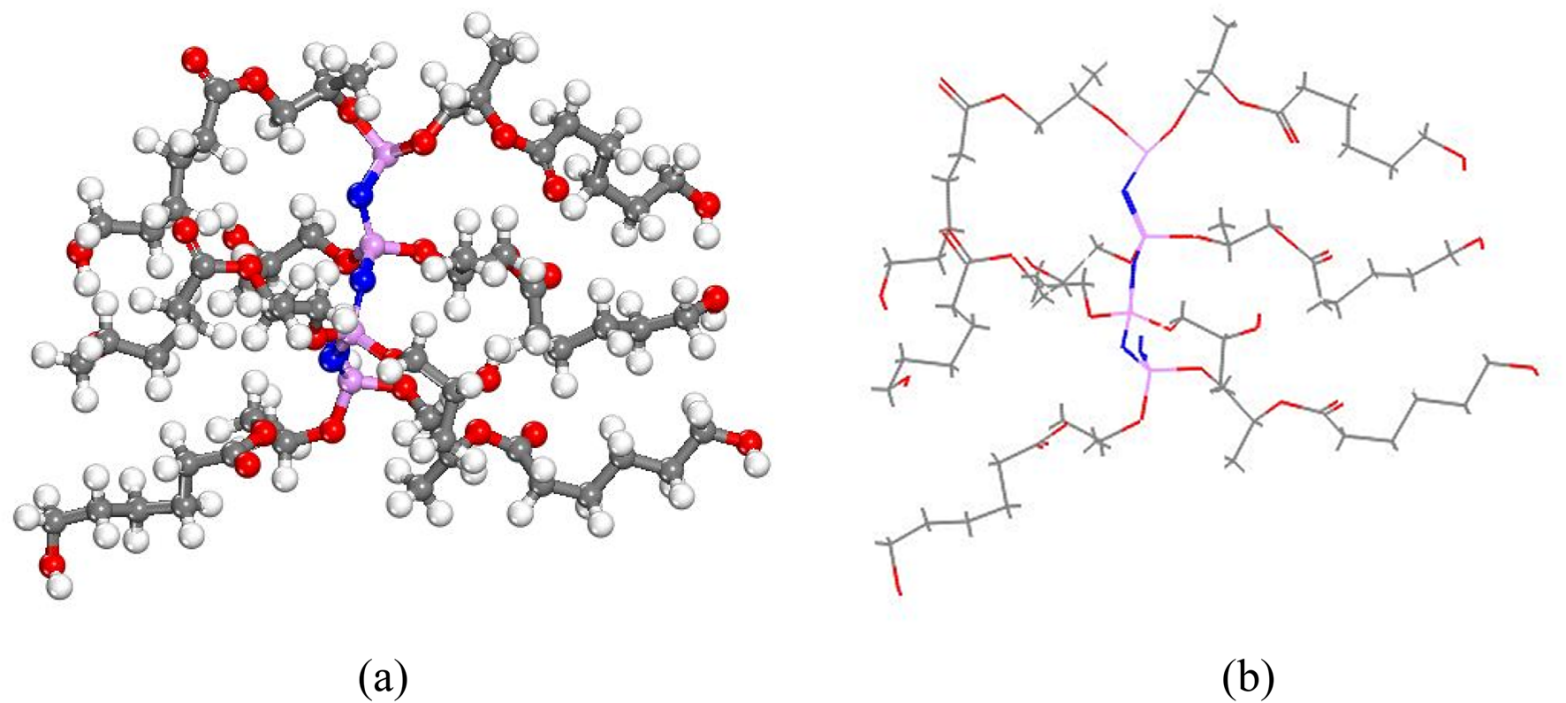
(**a**) and (**b**) The optimized structure of synthesized polymer molecule based on DFT-D correction by Materials Studio software2017.

The total energy value, atomic, Kinetic, Electrostatic, Exchange–correlation, and Spin polarization of polymer were obtained at − 6044.221, − 9613.762, 6009.122, − 43.960, − 15.126, 13.776, 10.530, and − 0.319 kcal/mol after optimization, respectively. The ball-and-stick model of optimized polymer is displayed in Fig. 8a,b.

**Figure 9:**
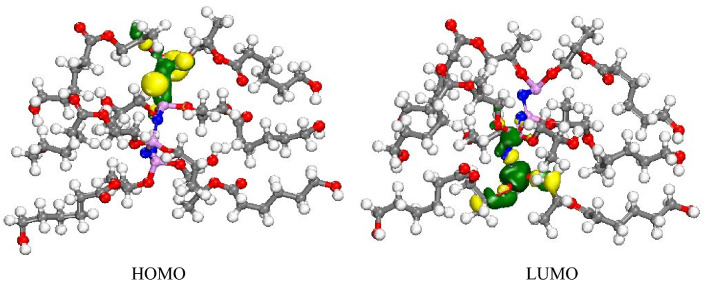
Schematic representations of HOMO and LUMO molecular orbitals of PPGP-*g*-PCL.

The HOMO and LUMO energies of polymer were calculated at about − 7.985 and − 0.560 eV, respectively. In addition, the electron charge of HOMO and LUMO orbitals of polymer distributed on phosphor and oxygen atoms linked to phosphor as well as methyl linked to an oxygen atom (Fig. 9).

### COSMO study of polymer

**Figure 10:**
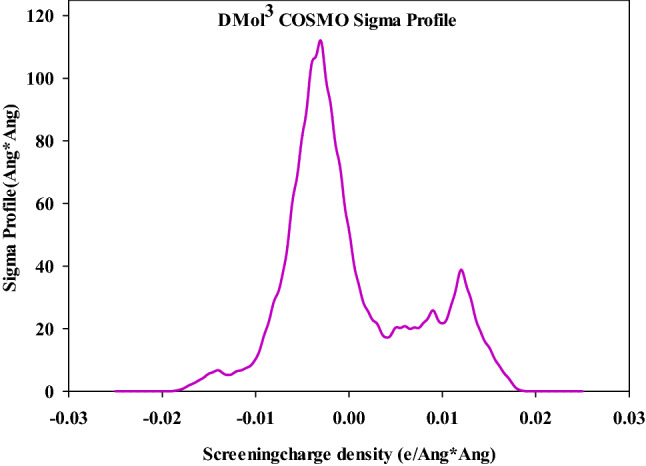
Sigma profile of the PPGP-*g*-PCL.

Figure 10 shows the σ-profile of polymer. The peaks located between 0 and + 0.01 e Å^−2^ can be related to the carbon of methyl groups or the non–polar nitrogen groups^40,41^. The peaks between 0 and − 0.01 e Å^−2^ in the σ-profile are related to the hydrogen of CH_3_ and CH_2_ groups. In addition, the peaks between + 0.010 to + 0.020 e Å^−2^ can be assigned to the O or N atoms^40,41^.

### Molecular dynamics simulations molecular

**Figure 11:**
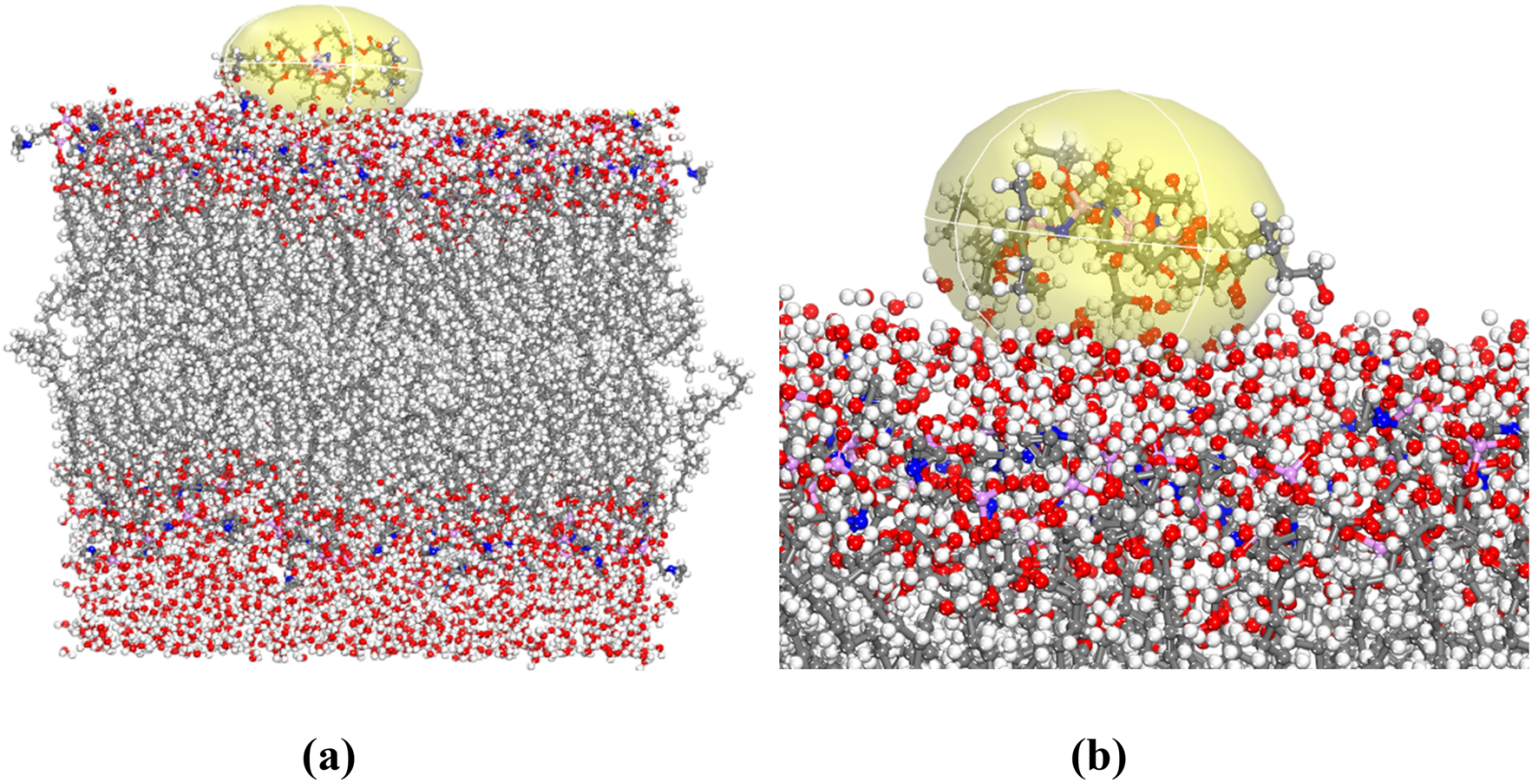
(**a**) and (**b**) Optimized geometries of fabricated PPGP-*g*-PCL adsorption on POPC surface.

The prepared simulation box, treated as periodic structures were optimized in the MS Forcite module along with charge; use current to obtain the total potential energy of the equilibrium state. The “SMART” algorithm with 50,000 steps was used for geometry optimization^42^. The adsorption energy (E_ads_) was calculated to achieve intermolecular interaction energy between polymer and POPC in the presence of water using Eq. (1) ^43^

1 $$\Delta {\text{E}}_{{{\text{ads}}}} = {\text{E}}_{{{\text{POPC}} - {\text{pol}} }} - \left( {{\text{E}}_{{{\text{POPC}}}} + {\text{E}}_{{{\text{pol}}}} } \right)$$

where E_POPC − pol_ is the total potential energy of the phospholipid membrane and polymer, E_POPC_ and E_pol_ are the total potential energy of the phospholipid membrane and synthesized polymer, respectively. The total energy of POPC, PPGP-*g*-PCL polymer and POPC-polymer were obtained 16,319.178, 31.756 and 141,148.658 kcal/mol, respectively. The optimized adsorption structure was represented in Fig. 11. In the liquid phase, the polymer adsorbed on the POPC during all simulation time. The free energy calculations of ΔE_ads_ were obtained significantly more negative (− 124,797.724 kcal/mol). This indicated that, at neutral pH in water, adsorption is a spontaneous process and is more favorable on POPC. The orientation of the PPGP-*g*-PCL molecule, parallel to the POPC surface, shows that it was adsorbed oriented interacting via strong negative. In addition, hydrogen bonding plays a significant role to adsorb the polymer on POPC surface.

### In vitro evaluations

#### Cell adhesion of PPGP-g-PCL

**Figure 12:**
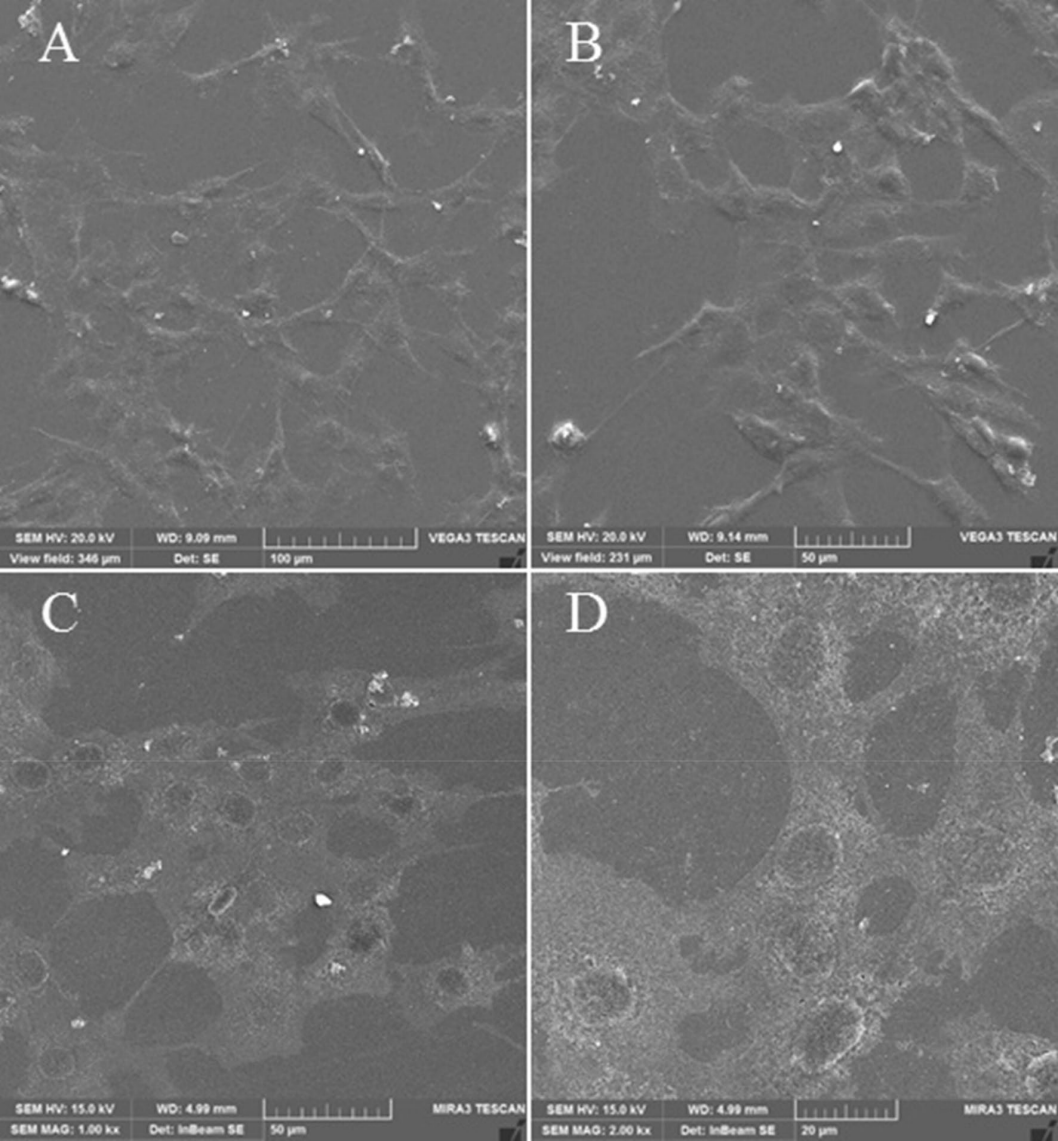
FESEM micrographs of adhered osteoblasts on the PPGP-*g*-PCL matrices surfaces 48 h after C2C12 culture at different magnifications: (**A**) 100, (**B**) 50, (**C**) 50, and (**D**) 20 µm.

Biocompatibility of PPGP-*g*-PCL was investigated qualitatively by FESEM when C2C12 myoblasts were cultured on it for 48 h. FESEM observations illustrated an excellent cell adhesion to the polymer and cell spreading, proliferate and differentiation throughout the surface (Fig. 12). Versus the PCL scaffolds often have weak surface bioactivities such as adhesion, osteoinduction, and growth of cells^2,40,44^. This is possible due to the hydrophobicity nature of PCL and the higher hydrophilic nature characteristics of PPGP-*g*-PCL, as confirmed in contact angle measurement.

#### In vitro cytotoxicity study

**Figure 13:**
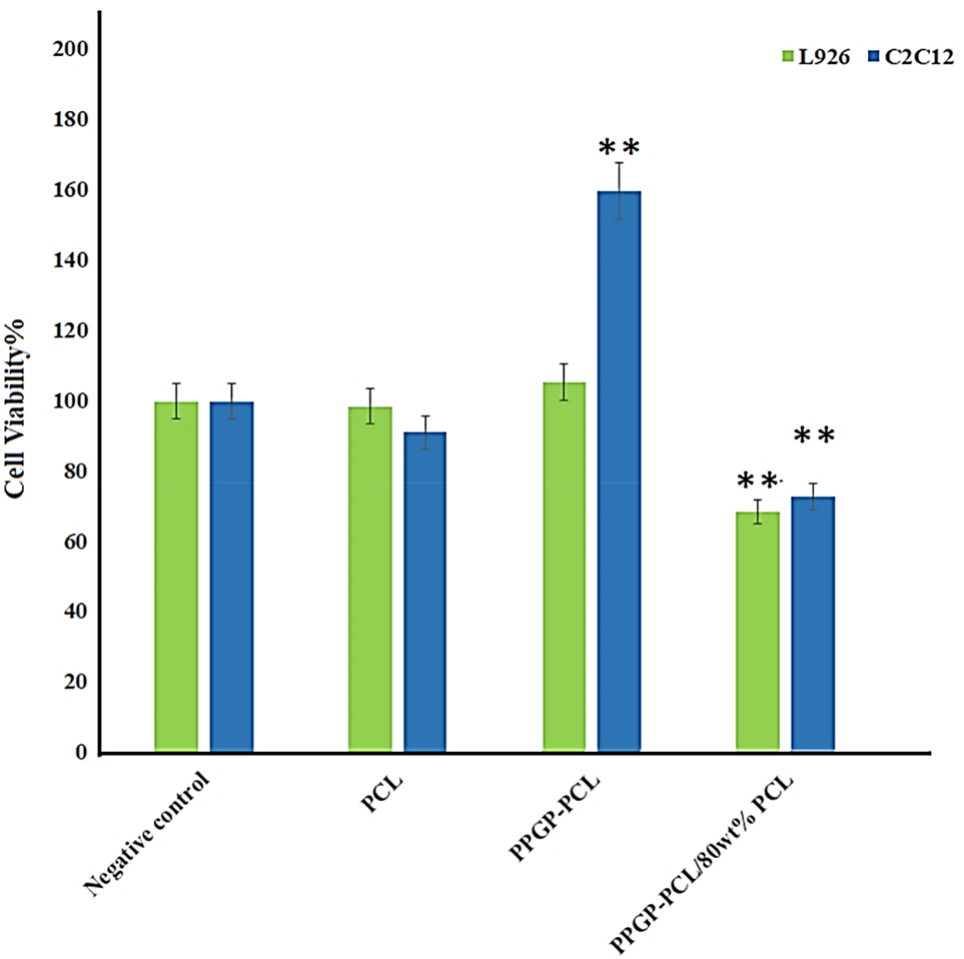
MTT assay of cells proliferation and viability on PPGP-*g*-PCL scaffolds and positive and negative controls during 72 h of culture. The data represent the means ± SD of the results from 3 replicates (n = 3). *P* < 0.05 versus control. Error bars represent means ± standard deviation.

Cell cytocompatibility for the cells cultured on three scaffolds, untreated PCL, PPGP-*g*-PCL, and blend polymer of PPGP-g-PCL/PCL (20/80 wt%), was evaluated using 3-(4,5-dimethylthiazol-2-yl)-2,5-diphenyltetrazolium bromide (MTT) assay at 72 h after cell seeding. According to Fig. 13, all scaffolds showed a high cell viability rate (higher than 90%). The results of the MTT cell-proliferation assay shows enhanced cell growth on PPGP-*g*-PCL and PPGP-*g*-PCL/PCL polymers compared to pure PCL. The cell viability of polymeric scaffolds (3 day) was significantly higher than the control groups (*P* < 0.05). In general, PPGP-*g*-PCL polymer presented the most suitable biocompatibility in all cells-seeded scaffold groups. The assay results clearly demonstrated that our synthetic scaffold was safe enough for biological application due to its non-toxic nature.

### In vivo evaluations

**Figure 14:**
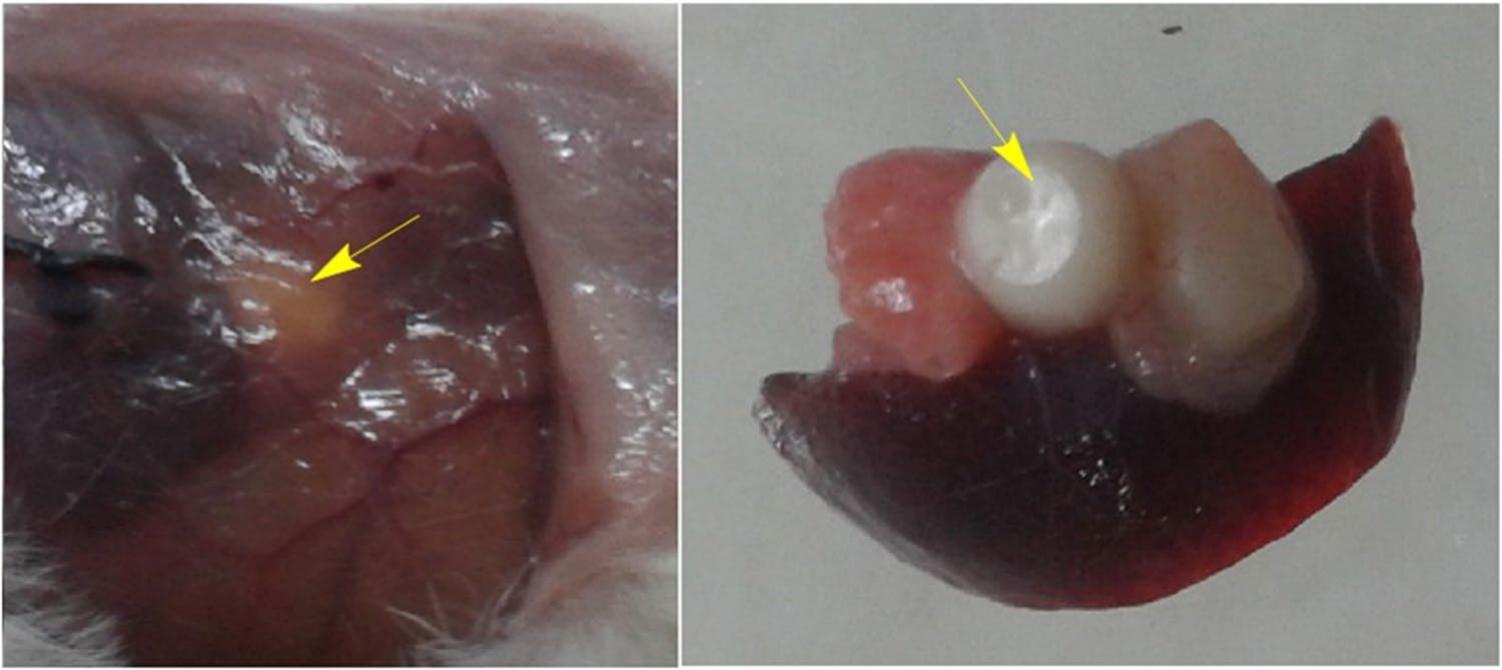
Photographs of mice 45 days after implantation intraperitoneally with PPGP-*g*-PCL/PCL scaffold (yellow arrows). The blood vessels and integration of the scaffold with animal tissues can be seen.

The scaffold's biocompatibility was also studied in 3 ± 4-week-old BALB/c mice using intraperitoneal implantation of conventional PCL and its mixture with PPGP-*g*-PCL new polymer and an untreated negative control group. None of the mice in the groups died within 45 days. During the experimental period, mice exhibited normal behavior in mobility, food intake, drinking water, nest building, … and any surgery-related or implantation-related complications were not observed. After mice sacrifice, macroscopic examination of tissue surrounding the scaffold did not show evidence of unnatural form or color, infection, or inflammation, as well as the scaffold, was merged with animal tissue and surrounded by blood vessels (Fig. 14).

**Figure 15:**
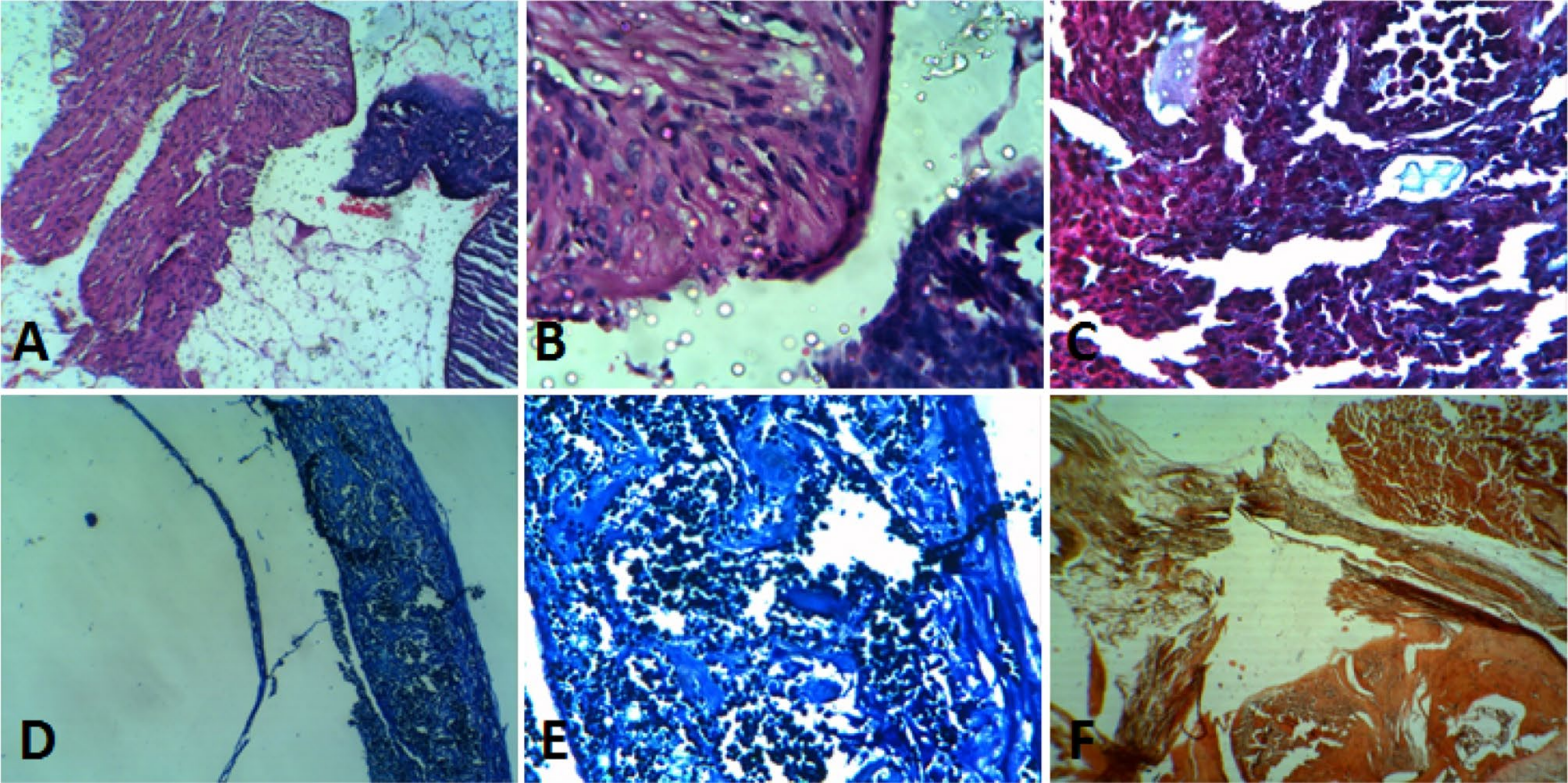
Results from the Histological evaluation of stained tissue slices by four types of staining when implanted with PPGP-*g*-PCL/PCL scaffold; (**A**): H-E stain at 100× magnification, (**B**): H-E stain at 400× magnification, (**C**): TRI stain at 400× magnification, (**D**): methylene blue stain at 100× magnification, (**E**): methylene blue stain at 400× magnification, and (**F**). alizarin red stain at 400× magnification.

When comparing the histopathological characteristics of organs adjacent to two implanted scaffolds, it was found that, despite having similar results for necrosis, inflammation, fibrosis, and fatty change (all 0 or close to 0), they have significant differences in the extent of newly formed bone, the osteoblast cells count and remaining scaffold percentage. Histomorphometric data are reported in Table 2 and various histological staining of segments are shown in Fig. 15A‒F. These observations indicate that the synthesized new polymer offer faster degradation properties, further cell conductivity, and significant tissue formation. The results of cell culture and in vivo comparison introduce PPGP-*g*-PCL as a non-toxic and biocompatible polymer.

**Table 2:** Data of histocompatibility evaluation after implantation of the synthetized polymer.

	PCL	Blend polymer
Necrosis	0	0
Inflammatory cells	+ 1	+ 1
Fibrosis	+ 1	+ 1
Fatty change	0	0
Extent of newly formed bone (%)	14	24
Numbers of osteoblasts (per 10 H.P.F)	9	16
Filling rate of defects lengths with newly formed bone (%)	11	20
Residual scaffold in the defect area (%)	36	23
MT	+ +	+ +
MB	+	+
AR	+	+ +

+ , Low.

+  + , Moderate.

## Conclusions

In this paper, we rendered evidence that displays the extent of the efficiency of PPGP-*g*-PCL polymer, synthesized via ROP of ε-caprolactone by PPGP multisite initiator, in tissue engineering applications. NMR, FTIR and EDS identified the structural and chemical specifications of synthesized compounds. It also investigated the surface morphology, thermal features, and hydrophobicity/hydrophilicity properties of the polymer using FESEM, TGA and DSC, and contact angle measurement, respectively. The molecular geometry and its energy, as well as HOMO and LUMO of poly(propylene glycol phosphazene)-*graft*-polycaprolactone (PPGP-*g*-PCL), was investigated using Perdew–Burke–Ernzerh (GGA/PBE) as well as Grimme method and Basis set; DND; 3.5 (similar to 6-311G*). HOMO and LUMO orbitals of polymer distributed on phosphor and oxygen atoms linked to phosphor and methyl linked to the oxygen atom. In order to understand the polymer adsorption on the phospholipid membrane, MD calculations were used to achieve total interaction energy in the presence of water molecules in material studio2017. The results of total adsorption energy values represented highly negative, suggesting more system stability. Results of in vitro and in vivo evaluations show that the PPGP porous scaffolds possess better cytocompatibility and outstanding biological behavior as compared with the pure PCL scaffold. These observations are due to the higher hydrophilicity of the novel polymeric scaffold, which results in more significant cell adhesion and proliferation. The study demonstrates that the grafting of the PCL onto hydroxylated polyorganophosphazene (PPGP), for fabrication of PPGP-*g*-PCL, can improve poor surface properties and inferior bioactivity of PCL.

## Materials and methods

### Materials

Ammonium heptamolybdate tetrahydrated, Aluminum chloride anhydrous (99.999%), Sodium sulfate anhydrous (≥ 99.0%), Hexachlorocyclophosphazene, Chloroform, Tetrahydrofuran (THF), Ethanol, Methanol, Zinc acetate dehydrate and Toluene were purchased from Sigma-Aldrich. Propylene glycol (99%), Paraformaldehyde, Dimethyl sulfoxide (≥ 99.9%) and ɛ-Caprolacton (ɛ-CL, 98%) were purchased from Merck. All chemicals used without further purification except when mentioned specifically. Phosphate-buffered saline (PBS) were purchased from Sigma-Aldrich. Sodium dihydrogen phosphate dehydrate, disodium hydrogen phosphate were purchased from Merck. All reagents for cell cultures were from Gibco. Plastics and tissue culture plates were from SPL, Korea. Applied cells were acquired from Pasteur Institute, Tehran, Iran.

### Physical and analytical method

Room temperature FT-IR spectra of synthetic samples were determined on a Perkin Elmer Spectrum RX I spectrophotometer using KBr pellets. ^1^H, ^13^C and ^31^P NMR spectra were carried out on a Bruker Avance DRX-500 spectrometer at room temperature. The X-ray diffraction (XRD) patterns were recorded using X’Pert PRO MPD X-ray diffractometer (Netherlands, Panalytical Company) for Cu-Kα radiation at 40 kV and 30 mA. The FE-SEM images of fabricated fillers, the morphology observation of adhered cells on the scaffold and energy dispersive spectra (EDS or EDX) of synthetic polymers were obtained by a TESCAN FESEM instrument model MIRA3TESCAN-XMU operating at 15 kV. The Thermogravimetric analysis (TGA) was performed from room temperature to 800 °C with 20 °C/min heating rate and under nitrogen atmosphere using a METTLER Thermogravimetric analyzer. The glass transition temperature (T_g_) was determined by differential scanning calorimetry (DSC) on a METTLER TOLEDO (Switzerland) with Thermal Analysis software. First polymer samples were dried in a vacuum oven at 50 °C. Then polymers (~ 10 mg) were heated from − 70 to 245 °C at a heating/cooling rate of 20 °C/min under a nitrogen atmosphere. The T_g_ was calculated by finding the half-height of the thermogram and determined in the second heating run after fast cooling. Water contact angle with the surface of synthetic polymeric scaffolds was measured using the sessile drop method of distilled water with a Rame’-Hart contact angle goniometer (model 100-00-220, USA). In vitro cell viability assay was evaluated by MTT assay at the wavelength of 570 nm by a spectrophotometric microplate reader (BioTek, MQX200 UQUANT USA).

### Synthesis of poly(propylene glycol)phosphazene (PPGP)

The PPGP polymer was synthesized and characterized similar to previously published articles and in the following way: 2 g of Hexachlorocyclophosphazene (HCCP), 4 g of propyleneglycole (in excess) and 0.04 g AlCl_3_ as the reaction catalyzer were added to a container equipped with a magnetic stir bar and sealed under inert gas. The reaction vessel was heated at 210 °C for three hours and then stirred at 90 °C for 18 h. The brown gel-like matter was solved in THF then filtered to separate AlCl_3_ and cross-linked polymers from synthesized PPGP. The solvent was evaporated, and the resulting pure polymer was dried in a vacuum oven at 60 °C overnight. This polymer dissolves well in water.

^31^P NMR of PPGP (500 MHz, DMSO-d_6_): δ = an almost broad peak at around − 1 ppm. ^1^H NMR (500 MHz, DMSO-d_6_): δ = 0.98–1.18 ppm (m, methyl), 3.24–3.77 ppm (m, methylene and methine), 4.14–4.17 ppm and 4.30–4.39 ppm (primary and secondary OH groups).

FT-IR (KBr): P–N (869 cm^−1^), P–O (1056 cm^−1^), P=N (1209 cm^−1^), Stretching vibration of C–H of CH_2_ and CH_3_ groups (2944, 2981 cm^−1^), bending vibration of CH_2_ and CH_3_ groups (1456 cm^−1^), bending vibration of methine (1339 cm^−1^), OH (br, 3400 cm^−1^).

### Synthesis of poly(propyleneglycol)phosphazene-graft-polycaprolactone (PPGP-g-PCL)

A 150 mL three-necked flask was equipped with a magnetic stirring bar, thermometer, an argon inlet and outlet tube, and a condenser. 1 g of PPGP polymer was dissolved in 20 ml toluene at room temperature. While stirring continuously, 0.01 g of zinc acetate dehydrate (Zn(OAc)_2_.2H_2_O) and 0.001 g of p-methoxyphenol were added. The resulted mixture was stirred for 15 min until all materials were solved entirely. Then, 1 g *ɛ*-caprolacton was added, and the resulted solution was refluxed under inert gas for six hours at a temperature 115 °C. The final product was obtained by complete removal of toluene in 80 °C. A wax-like brown substance remained that, for further purification, was dissolved in THF and recovered by evaporation of THF under vacuum in 50 °C. Resulted polymer is well soluble in ethanol and insoluble in water.

^31^P NMR (500 MHz, CDCl_3_-d_1_,): an almost broad peak at around δ =  − 1 ppm.

^1^H NMR (500 MHz, CDCl_3_-d_1_): δ = 1.04–1.18 ppm (CH_3_), 1.29–1.40 ppm (m, CH_2_), 1.51–1.67 ppm (m, CH_2_), 2.25 ppm (t, CH_2_), 3.57 ppm (t, CH_2_), 3.66 ppm (d, CH_2_), 3.88–3.93 ppm (m, CH), 4.00 ppm (t, CH_2_).

^13^C NMR (500 MHz, CDCl_3_-d_1_): δ = 173.5 ppm (C=O), 69.41, 68.22 ppm (CH), 67.90, 65.84, 64.08, 62.38, 34.07, 32.2, 28.9, 25.49, 24.50 ppm (CH_2_), 19.24, 16.70 ppm (CH_3_).

FTIR (KBr): 3442.31 cm^−1^ (OH), 2942.62 cm^−1^ and 2865.37 cm^−1^ (Stretching vibration of CH_2_ and CH_3_), 1726.46 cm^−1^ (C=O), 1460.31 cm^−1^, 1419.02 cm^−1^ and 1371.59 cm^−1^ (bending vibrations of CH_2_ and CH_3_), 1295.37 cm^−1^ (C–O and C–C stretching), 1244.34 cm^−1^ (C–O–C stretch asymmetric), 1193.25 cm^−1^ (P=N), 1166.38 cm^−1^ (C–O–C), 1091 cm^−1^ (P–O), 1166.38 cm^−1^ (C–O–C).

### Synthesis of polycaprolactone (PCL)

We used PCL as the positive control to biological evaluations of synthesized polymer and also to prepare the blend polymers. For CL polymerization, 1 g *ɛ*-Caprolacton, 0.005 g zinc acetate dehydrate (Zn(OAc)_2_·2H_2_O), and 0.002 g p-methoxyphenol were added to 5 ml anhydrous toluene in a three-neck round-bottom flask. The system was equipped with a condenser for refluxing, a magnetic stirring bar, and a thermometer. After materials were completely dissolution, the reaction was refluxed for six hours, and then toluene was removed under reduced pressure at 70 °C. Afterward, remained product was resolved in THF, centrifuged at 8000 g, and the supernatant was under vacuum at 40 °C to remove THF. The obtained polymer is insoluble in ethanol.

^13^C NMR of PCL (500 MHz, CDCl_3_-d_1_): δ = 173.4 ppm (C=O), 64, 62.4, 34, 32.1, 28.2, 25.4, 24.5 ppm (CH_2_), 18 ppm (CH_3_).

IR of PCL (KBr): 3439.82 cm^−1^ (OH), 2950.45 cm^−1^ and 2866.83 cm^−1^ (CH_2_), 1728.47 cm^−1^ (C=O), 1471 cm^−1^, 1419 cm^−1^ and 1366 cm^−1^ (CH_2_ bending vibrations), 1294 cm^−1^ (C–O and C–C stretching) 1240.44 cm^−1^ (C–O–C stretch asymmetric), 1172.86 cm^−1^ (C–O–C).

### Mechanical properties and degradability behavior of the fabricated scaffolds

Compressive mechanical testing was accomplished using a universal testing device (Santam, Karaj, Iran) to valuate compressive strength. The fabricated pieces of each scaffold (height of 20 mm and diameter of 7 mm) were placed and the compression load was exerted at a crosshead rate of 1 mm/min.

The in vitro degradation experiments were carried out on PCL, PPGP-g-PCL and PCL/PPGP-g-PCL polymers to compare the degradability of all scaffolds. The samples were attentively made into 5 mm × 5 mm × 2 mm dimensions, weighed, and located in glass test vials. Two medium were ready for degradation studies: phosphate-buffered saline (PBS) (pH 7.4) and acetate sodium/acetic acid (AcOH) buffer (pH 4.2). The specimens were submerged in the solutions and incubated at 37 ˚C. At the preset timeouts, the samples were removed from the media and weighed after freeze-dried. The degradation rate were calculated using Eq. (1).

2 $${\text{Degradation}}\; \left( {{\text{rate of weight loss}} \% } \right) = \frac{{W_{t} - W_{i} }}{{W_{i} }} \times 100\%$$

wherever “W_i_” is the initial weight of instance and “W_t_” is the dried weight at time t. Eventually, the degradation visual view was assessed by SEM imaging during the test period.

### Computational method

The quantum calculation was computed using by DMol^3^ module based on DFT-D correction to determine molecular geometry and its energy and HOMO and LUMO in Materials Studio software2017. These calculations were completed with the functional; generalized gradient approximation and the local density approximation (LDA) of Perdewe Wang (PWC) method and Basis set; DND; 3.5 (similar to 6-311G^*^). The solvation model was used to give sigma a profile, as a result, a smearing of 0.005 and a global orbital cutoff of 3.3°A at room temperature (298 K) were used^41,45^.

### Molecular dynamics simulations

Pdb (POPC128b) of phospholipid membrane having 128 POPC lipids and 2460 water molecules was downloaded from https://people.ucalgary.ca/~tieleman/download.html, and POPC128b is shown in (Fig. S2). Then, in order to MD simulations, the lattice parameters of bilayer topology (shown in Fig. 8) and polymer were improved based on (010) surface and thickness of 1.55 Å with a = 62.391, b = 61.797, c = 99.960 and, α = β = γ = 90.000°. Afterward, POPC, polymer and POPC- polymer were run in Materials Studio at room temperature (298 K) using Forcite calculation; Task; Geometry optimization, electrostatic and van der Waals; Group based as well as the Universal force field to obtain energy.

### Biological evaluations

#### Adhesion and proliferation of cell

In this study, C2C12 and L929 cell lines were used as the model cells. In order to in vitro cytotoxicity test, the scaffolds were prepared with PPGP-*g*-PCL alone, and a physical mixture of PPGP-*g*-PCL (20%W) with the polymers pure PCL (80%W). The PCL, the most studied scaffold, alone was also used as a positive control. The scaffolds were placed into the 96-well polystyrene plate, sterilized by gentamicin and washed by sterilized PBS three times. C2C12 and L929 cells lines were subcultured, trypsinized and then seeded on treated scaffolds at a density of 3 × 10^4^ cells/well and then were maintained at 37 °C, 5% CO_2_ in a humidified incubator. DMEM (Dulbecco's Modified Eagle Medium) supplemented with 5% FBS (Fetal Bovine Serum) and 1% PS (Penicillin and Streptomycin) was used as cell culture medium. After culture for three days, the culture medium was replaced with culture media containing 50 µl of MTT solution and was placed in a 37 °C incubator with 0.5% CO_2_ for 4 h. After incubation, the excess amount of MTT was removed and, for dissolve the Formazan crystals were refilled with 200 µl of DMSO. The color exchange was quantified spectrophotometrically at 570 nm.

### Electron microscopy analysis of cultured cells

Field emission scanning electron microscopy (FESEM) was used to observe the shape and morphology of cultured cells. PPGP-*g*-PCL scaffolds containing C2C12 cells were first washed with PBS three times and then were fixed by immersing in PBS containing 4% paraformaldehyde (pH 7.4) for 30 min. The cell/scaffold constructs were again rinsed with PBS and dehydrated in a series of increasing ethanol concentrations (50‒100%) in each of the concentrations for 10 min. After thoroughly dried, the samples were coated with gold for microscopic observation by FESEM.

### In vivo biocompatibility

Miscible polymers of PCL and PPGP-*g*-PCL in a weight ratio of 80:20 with a total weight of 1 g were mixed at 50 °C to obtain a homogeneous mixture. At 50 °C temperature, the viscosity of the resulting blend polymer (PCL/PPGP-*g*-PCL) and pure PCL are appropriate to handling, therefore pieces with dimensions of 1.5 mm * 5 mm * 5 mm of pure PCL and a PCL/PPGP-*g*-PCL blend were carefully fabricated. Obtained pieces were cooled to ambient temperature to make tightening scaffolds and were sterilized by exposing them to UV light for 30 min.

Albino BALB/c mice (n = 5) 3‒4 weeks of age were used in this study and provided by the Pasteur Institute of Iran. According to international guidelines on the use of laboratory animals, the animals were cared for and approved by the Ethical Committee of Tarbiat Modares University (Ethics board approval number: IR.MODARES.REC.1397.079) and followed the ARRIVE guidelines (PLoS Bio 8(6), e1000412,2010). The mice were anesthetized with a combination of Ketamine and Xylazine, and all efforts were made to minimize suffering. For in vivo testing, scaffolds include PCL/PPGP-*g*-PCL blend (80/20) and only PCL, as control, were implanted in the peritoneal cavities. Forty-five days after intraperitoneal implantation, the examined animals were killed humanely. Next, the implants and the surrounding tissues were removed and immediately fixed in buffered formalin. Fixed sections were processed for analysis of any tissue reaction or inflammation using Hematoxylin and Eosin (H-E) and Masson's trichrome (MT) stains. Calcium content change and amount of mineralization in tissue were confirmed via Alizarin Red staining. Then, formalin-fixed tissues were stained with methylene blue stain to distinguish other types of calcification of tissue. All stained sections were fixed onto the glass slides and then examined microscopically by a pathologist. The pathologist also analyzed the histological parameters by a computer software.

### Statistical analysis

Data were expressed as means ± standard deviation (SD) of three independent experiments and were presented relative to control. Data were analyzed by one-way analysis of variance (ANOVA). Statistical comparison between treatment groups (experimental and control) was performed by Student’s t-test using R Statistical Software (version 3.5.1). A *P*-value < 0.05 is defined statistically significant difference, and some values were accepted.

## Supplementary Information

Supplementary Information.

## Data Availability

The datasets generated during and/or analyzed during the current study are available from the corresponding author on reasonable request.
