# Supplementary material for: Fabrication and examination of polyorganophosphazene/polycaprolactone-based scaffold with degradation, in vitro and in vivo behaviors suitable for tissue engineering applications
Source: Sci Rep. 2022 Nov 1;12:18407. doi: 10.1038/s41598-022-18632-8 (PMC9626536; doi:10.1038/s41598-022-18632-8)
Supplement: Supplementary file 1 — Supplementary Information. [file 41598_2022_18632_MOESM1_ESM.docx]

Fabrication and examination of polyorganophosphazene/polycaprolactone-based scaffold with degradation, in vitro and in vivo behaviors suitable for tissue engineering applications

Khodayar Gholivand, *^,a,†^ Mahnaz Mohammadpour, ^a,†^ Seyed Alireza Alavinasab Ardebili, ^a^ Rahime Eshaghi Malekshah ^b^ and Hadi Samadian ^c,†^

^a^ Department of Chemistry, Faculty of Sciences, Tarbiat Modares University, Tehran, Iran.

^b^ Department of Chemistry, Iran University of Science and Technology, Tehran, Iran.

^c^ Department of Molecular Medicine, School of Medicine, Hamedan University of Medical Sciences, Hamedan, Iran.

^*^ Corresponding author’s E-mail address: [Gholi_kh@modares.ac.ir](mailto:Gholi_kh@modares.ac.ir)

^†^ These authors contributed equally to this manuscript.


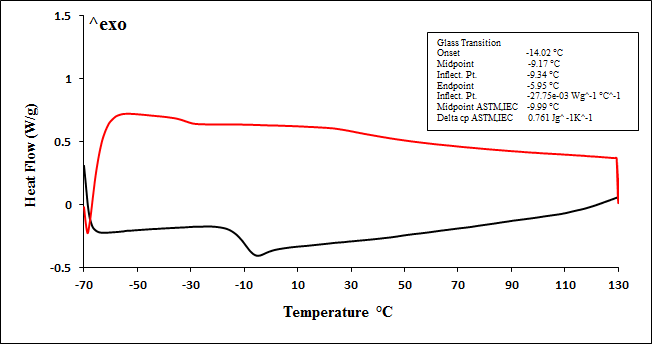


**Figure. S1.** DSC thermogram of PPGP (first heating (black) and cooling (red)).

| 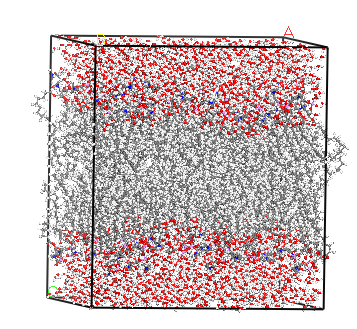 | 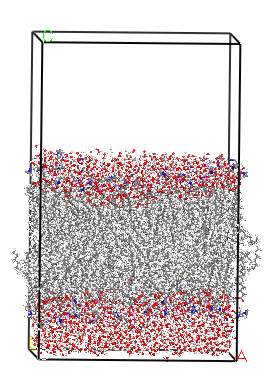 |
| --- | --- |
| a | b |

**Figure. S2.** Snapshot of (a); phospholipid membrane (pdb; POPC128b) and (b); phospholipid membrane unit cell on (010) surface.


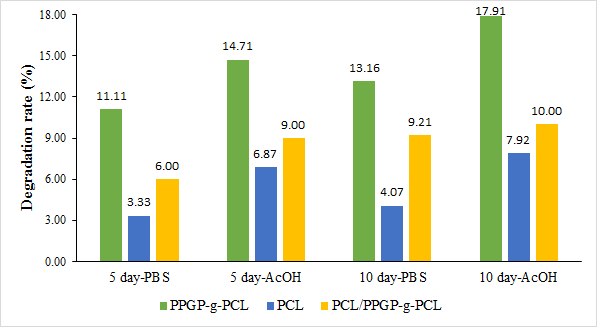


**Figure. S3.** Biodegradation profile of three kinds of fabricated scaffolds as a function of immersion time in (A) AcOH buffer and (B) PBS during 10 days.


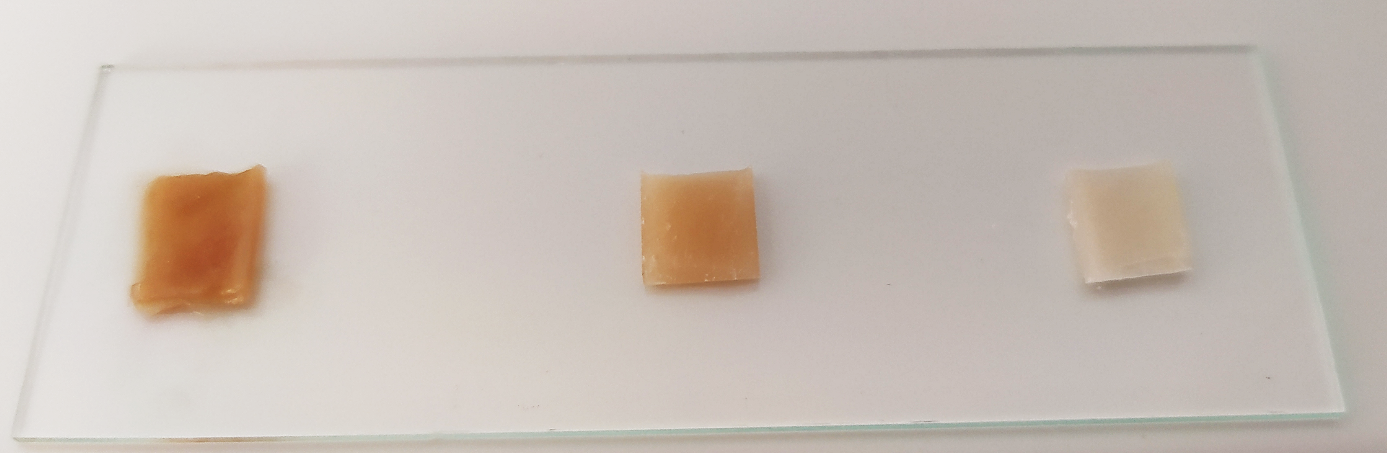


**PPGP-g-PCL PCL/PPGP-g-PCL PCL**

**Figure. S4.** Representative picture of fabricated scaffolds, without any treatment, in approximate dimensions of 5 mm * 5 mm * 1.5 mm.
